# High prevalence and genetic diversity of *Haemoproteus columbae* (Haemosporida: Haemoproteidae) in feral pigeons *Columba livia* in Cape Town, South Africa

**DOI:** 10.1007/s00436-019-06558-6

**Published:** 2019-12-27

**Authors:** Carina Nebel, Josef Harl, Adrien Pajot, Herbert Weissenböck, Arjun Amar, Petra Sumasgutner

**Affiliations:** 1 FitzPatrick Institute of African Ornithology, DST-NRF Centre of Excellence, University of Cape Town, Cape Town, South Africa; 2 Institute of Pathology, University of Veterinary Medicine, Vienna, Austria; 3 Bordeaux Sciences Agro, 1 Cours du Général de Gaulle, Gradignan, France; 4 Konrad Lorenz Forschungsstelle, Core Facility for Behaviour and Cognition, University of Vienna, Vienna, Austria

**Keywords:** Haemosporidian parasites, Southern Hemisphere, Microscopy, Blood smear, Mitochondrial *cytochrome b*, Co-infection, Pleiotropic effects—eumelanin, Urbanization, Urban ecology

## Abstract

In this study, we explore blood parasite prevalence, infection intensity, and co-infection levels in an urban population of feral pigeons *Columba livia* in Cape Town. We analyze the effect of blood parasites on host body condition and the association between melanin expression in the host’s plumage and parasite infection intensity and co-infection levels. Relating to the haemosporidian parasite itself, we study their genetic diversity by means of DNA barcoding (*cytochrome b*) and show the geographic and host distribution of related parasite lineages in pigeons worldwide. Blood from 195 *C. livia* individuals was collected from April to June 2018. Morphometric measurements and plumage melanism were recorded from every captured bird. Haemosporidian prevalence and infection intensity were determined by screening blood smears and parasite lineages by DNA sequencing. Prevalence of *Haemoproteus* spp. was high at 96.9%. The body condition of the hosts was negatively associated with infection intensity. However, infection intensity was unrelated to plumage melanism. The *cytochrome b* sequences revealed the presence of four *Haemoproteus* lineages in our population of pigeons, which show high levels of co-occurrence within individual birds. Three lineages (HAECOL1, COLIV03, COQUI05) belong to *Haemoproteus columbae* and differ only by 0.1% to 0.8% in the *cytochrome b* gene. Another lineage (COLIV06) differs by 8.3% from the latter ones and is not linked to a morphospecies, yet. No parasites of the genera *Leucocytozoon* and *Plasmodium* were detected.

## Introduction

Wild feral pigeon populations of *Columba livia* f. *domestica* Gmelin, 1789 are typical inhabitants of urban landscapes, where they are associated with humans due to the availability of suitable nesting sites and anthropogenic food. These factors have enabled feral pigeons and many of its parasites to colonize cities across the globe (Johnston and Janiga 1995). The undomesticated relative is the rock pigeon *Columba livia*, whose native range is restricted to Western and Southern Europe. Archeological evidence suggests that rock pigeons were domesticated several thousand years ago (Shapiro and Domyan 2013) and have been used as a food source as early as 10,000 years ago (Blasco et al. 2014). Since then, feral pigeons have successfully expanded their range and established wild populations worldwide, mainly in urban environments. The feral pigeon is not native to South Africa but was introduced as a domestic species in the seventeenth century (Brooke 1981; Dean 2000). Cape Town is a hotspot of biodiversity (Myers et al. 2000) and of special interest as an urban study location. Not only is the literature body comparatively scarce in the “Global South” in comparison to the “Global North” (Hedblom and Murgui 2017), but urban environments are also rapidly changing in the Southern Hemisphere (Swilling 2006). Feral pigeons are likely the most successful avian urban exploiters that manage very well in urban and suburban environments. Furthermore, they are also a model organism in many research disciplines, i.e., parasitology, behavioral science, and physiology (i.e., Abs 1983; Harbison et al. 2009; Klopfleisch et al. 2006).

Avian haemosporidians are comprised of apicomplexan protozoans of the genera *Plasmodium*, *Haemoproteus*, and *Leucocytozoon*. Haemosporidians are the most diverse group of avian blood parasites with several hundred species infecting birds all over the world (Valkiūnas 2004). The genus *Haemoproteus* contains two subgenera: *Haemoproteus* and *Parahaemoproteus* (Valkiūnas 2004). *Haemoproteus* comprises about ten parasite species described from Columbidae hosts and one species each from Laridae and Fregatidae hosts. *Parahaemoproteus* comprises the majority of *Haemoproteus* parasite specialized on passerines (Levin et al. 2012; Levin et al. 2011; Valkiūnas 2004; Valkiūnas et al. 2013; Valkiūnas et al. 2010). The two *Haemoproteus* subgenera form distinct clades in molecular phylogenetic analyses (Galen et al. 2018; Pacheco et al. 2017) and depend on different dipteran groups as vectors. Species of the subgenus *Haemoproteus* are transmitted by dipterans of the family Hippoboscidae, whereas species of the subgenus *Parahaemoproteus* are transmitted by biting midges of the family Ceratopogonidae (genus *Culicoides*).

*Haemoproteus columbae* Kruse, 1890 was described from *C. livia* and is transmitted horizontally by blood-sucking insects, predominantly by the ectoparasitic pigeon louse fly *Pseudolynchia canariensis* Macquart, 1839 (Sol et al. 2000). Blood stages of *Haemoproteus* species are quite conspicuous in blood smears (Valkiūnas 2004), but species determination is complicated due to the limited number of distinct morphological features. More recently, DNA sequencing using PCR has been established to identify blood parasite lineages (Fallon et al. 2003) and has also been used as a more accurate method for identifying blood parasite prevalence, especially at low infection intensities (Fallon and Ricklefs 2008; Fallon et al. 2003; Garamszegi 2010). Furthermore, genetic sequencing enables the identification of closely related blood parasite lineages, which cannot be differentiated through microscopic examination. Sequencing also allows the determination of co-infections of multiple haemosporidian lineages, which commonly occur (Alizon et al. 2013; Poulin and Morand 2000; Silva-Iturriza et al. 2012). To distinguish avian haemosporidian species or lineages, the mitochondrial *cytochrome b* (*cytb*) is often used as a genetic marker as it incorporates the so-called “DNA barcode” region for haemosporidians (Bensch et al. 2009; Dimitrov et al. 2014). Single and multiple infections of several blood parasite lineages can have different effects on the hosts and have been associated with negative effects on their body conditions (Marzal et al. 2008), lower hematocrit values (Palinauskas et al. 2011), and lower host survival (Pigeault et al. 2018). However, in studies on house martins *Delichon urbica*, birds with double infections showed better reproductive performance (Marzal et al. 2008; Pigeault et al. 2018).

Generally, *H. columbae* infections are not considered to be very detrimental to their hosts (Bennett et al. 1993; Earle et al. 1993; Sol et al. 2003). However, high infection intensity can be associated with negative physiological (Earle et al. 1993) and behavioral (Markus and Oosthuizen 1972) changes. In some cases, infections can even be lethal, especially in young individuals that have not yet developed an adequate immune response (Sol et al. 2003). Due to their successful expansion across the globe, feral pigeons act as vectors for *H. columbae* and other haemosporidian parasites. They can facilitate the spread into naïve new hosts and can potentially threaten local columbid bird species (Chagas et al. 2016; Foronda et al. 2004; Lee-Cruz et al. 2016; Peirce et al. 1997; Swinnerton et al. 2005; van Riper et al. 1986). Specifically in Cape Town, feral pigeons regularly form mixed flocks with the native speckled pigeon *Columba guinea* and might allow the spread of feral pigeon–specific blood parasites to naïve hosts (Earle and Little 1993; Little 1994). In an earlier study, *Haemoproteus* has been identified in speckled pigeons; however, this study was based on blood smear analysis only. In the absence of DNA sequencing, it remains unknown whether these represent blood parasite lineages originating from feral pigeons or whether they are unique to native speckled pigeons. Furthermore, feral pigeons were identified as potential vectors of novel parasites for endemic and endangered columbid species native in the Canary Islands (Foronda et al. 2004). *Haemoproteus columbae* has not yet been found in a non-columbid host and might be specific to pigeon and dove hosts (Valkiūnas 2004).

The domesticated feral pigeon is color polymorphic, whereby different morphs are independent of age or sex, are interbreeding freely, and can be present within the same population (Huxley 1955). The species features a wide range of plumage colorations ranging from completely white to almost fully melanistic (Haase et al. 1992; Jacquin et al. 2011, 2013; Johnston and Janiga 1995). Plumage phenotypes are determined by eumelanin and pheomelanin that are known for their pleiotropic effects on the immune system through the melanocortin system (Ducrest et al. 2008), whereby the gene coding the phenotype also controls the expression of several different traits, unrelated to color, such as immunocompetence. Plumage variation in the feral pigeon is associated with a stronger immune system (Jacquin et al. 2011), and white pigeons (i.e., lacking melanin) were found to have a higher blood parasite prevalence with increasing levels of urbanization (Jacquin et al. 2013). In turn, darker individuals showed a stronger cortisone stress response in rural areas than in urban habitats (Corbel et al. 2016).

In the present study, (1) we describe *H. columbae* prevalence and infection intensity in a wild feral pigeon population in the city of Cape Town, South Africa. Additionally, (2) we assess blood parasite diversity by PCR screening for *H. columbae* lineages and by sequencing a section of the mitochondrial *cytb* gene. (3) We show the geographic and host distribution of *H. columbae* and related parasite lineages in pigeons worldwide. (4) We test for potential health consequences of blood parasite infection by exploring whether higher infection intensity and co-infection with different *Haemoproteus* lineages reduce individual body condition. We hypothesize that those blood parasites have a negative effect on the health of the host and thus predict that individual body condition decreases with increasing infection intensity or infection by multiple parasite species. Lastly, (5) we explore for potential pleiotropic effects of color polymorphism by correlating infection intensity and co-infection with the expression of eumelanin. Here, we hypothesize that eumelanin expression influences an individual’s health. Therefore, we predict that feral pigeons with darker plumage coloration have lower haemosporidian infection intensity and a lower number of co-infections.

## Material and methods

### Study site and feral pigeons

For this study, 195 wild feral pigeons were caught in walk-in traps in suburban areas of Cape Town, South Africa (33.92° S, 18.42° E) from April to June 2018, during the austral autumn and winter. We attempted to obtain data for all birds on morphometric measurements, blood samples for smears, and DNA extraction; however, some data is missing from some birds due to time constraints in processing several individuals in a short period of time. Sample sizes (*n*) are given in brackets below throughout; for a complete sample list, see Appendix Table S1.

Before blood sampling, pigeons were routinely housed communally in a pigeon coop at Eagle Encounters, Stellenbosch (33.97° S, 18.78° E), for a maximum of 6 days prior to being part of an unrelated behavioral experiment (Nebel et al. 2019). A maximum of 1 ml blood was taken from the brachial vein of each individual, and these blood samples were then immediately use to prepare blood smears on microscope slides and air-dried. Remaining blood was stored in EDTA buffer at 4 °C. We measured the tarsus length with a caliper (to the closest 0.1 mm) and weighed the pigeon on a scale (in g). These measures were taken to derive individual body condition and were obtained from 187 individuals in total. The age of the pigeons was classified depending on the color of the eyes and cere (immature, < 6 months of age, *n* = 12, or adult, > 6 months, *n* = 183; following Kautz and Seamans 1986). Sex was not determined. All pigeons were individually marked and released back at the source of capture.

### Assessment of plumage phenotype

Plumage coloration in the feral pigeon is determined by eumelanin and pheomelanin deposited in the feathers (Haase et al. 1992). Although both eumelanin and pheomelanin are associated with the melanocortin system, and both can have beneficial effects on the immune system (Ducrest et al. 2008; Jacquin et al. 2011), in this study, we focus on pigeons showing a variation of eumelanin and excluded all individuals with a pheomelanin-based plumage (i.e., reddish plumage; *n* = 3) due to the small sample size. Systematic photographs were taken (showing the belly, back with one wing spread and the head) to document the plumage of every individual. The feral pigeons’ phenotypes were determined in a twofold approach. First, melanin content of the wing was quantified by using the software ImageJ (Abramoff et al. 2004) and then categorized according to levels of melanism by author AP following established protocols of Johnston and Janiga (1995) and Jacquin et al. (2011): phenotypes 0 (“white,” *n* = 1), 1 (“blue bar,” *n* = 40), 2 (“checker,” < 50% dark feathers on the wing surface, *n* = 57), 3 (“T-pattern,” > 50% dark feathers on the wing surface, *n* = 65), and 4 (completely melanistic, *n* = 28). The plumage of one pigeon could not be scored, because no photos were taken during processing. One individual showing mainly white feathers (score 0) was excluded from all analyses due to low sample size. Ten percent of all individuals were randomly re-scored, and the assignment was highly reproducible.

### Assessment of infection intensity by blood slide screening

Blood smears of 192 individual *C. livia* were fixed with methanol and stained with Giemsa stain following the standard protocol of Hemacolor^®^ Rapid staining of blood smear kit (Merck, Darmstadt, Germany). The slides were first scanned under the microscope at × 400 magnification to determine the presence or absence of haemosporidian blood parasites. For infected individuals, the intensity of infection was then determined by scanning each slide at × 1000 magnification with an oil immersion lens and counting the number of parasites seen within 10,000 erythrocytes.

### Molecular genetics: assessment parasite lineages and co-infections

Molecular screening for blood parasites and identification of *Haemoproteus* lineages were performed on 144 feral pigeon samples of which aliquots of blood were stored in EDTA buffer (in 51 cases, all obtained blood was used up, preparing the blood smears). DNA was extracted with the DNeasy Blood & Tissue Kit (Qiagen, Venlo, Netherlands) following the standard protocol for isolation from blood and using equivalents of about 10 μl blood from each sample. We amplified an 886-bp section of the mitochondrial *cytb* using the primers CytB_HPL_intF1 (5′-GAGAATTATGGAGTGGATGGTG-3′) and CytB_HPL_intR1 (5′-ATGTTTGCTTGGGAGCTGTAATC-3′) (Harl et al. 2019), which covers the so-called DNA barcode region for avian haemosporidians. CytB_HPL_intF1 binds at nucleotide positions 174 to 195 of the *cytb*, and CytB_HPL_intR1 binds at a conserved site 13-bp from the 3′ end of the *cytb*. PCRs were performed with the GoTaq Long PCR Master Mix (Promega, Madison, Wisconsin, USA) on a peqSTAR 2X Universal Gradient thermocycler (VWR, Radnor, Pennsylvania, USA). Each 25 μl master mix contained 12.5 μl GoTaq Long PCR Master Mix, 9.5 μl H_2_O, each 1 μl of 10 mM primer, and 1 μl of DNA template. The PCRs started with an initial denaturation for 2 min at 94 °C, followed by 35 cycles with 30 s at 94 °C, 30 s at 53 °C, 1 min at 65 °C, and a final extension for 10 min at 72 °C. PCR products were visualized on 1% agarose gels, and positive samples were purified with the QIAquick PCR Purification Kit (Qiagen). Sequencing was performed at Microsynth Austria GmbH (Vienna, Austria) using the PCR primer CytB_HPL_intR1. Raw sequences were inspected by eye with BioEdit v.7.0.8 (Hall 1999). Sequences of samples with double and triple infections featured ambiguous sites with wobble bases, which were consequently checked in the electropherograms. After the first inspection, the sequences were sorted by similarity and all aberrant sites were rechecked. Identification (and unphasing) of parasite lineages in samples with multiple infections was straightforward, because the number of different haplotypes in our sample was low. Haplotype networks, based on a 478-bp *cytb* fragment, were calculated with two different datasets, the first comprising haplotypes related to *H. columbae* and the second comprising the lineage of a yet unidentified *Haemoproteus* species (COLIV06) and related haplotypes. To visualize the geographic and host distribution, we included already published sequences from GenBank (NCBI) in the networks. These were retrieved by performing BLAST searches and including all samples with sequence similarity of 97% to 100% to haplotypes found in the present study. Only those BLAST hits were kept, which did not contain ambiguities and which covered the 476-bp DNA barcode region of the *cytb*. The respective lineage names of the haplotypes were retrieved by BLAST searches against the MalAvi database (Bensch et al. 2009). The sequences were aligned with MAFFT v.7 (Katoh and Standley 2013) and trimmed to the size of the barcode region. Median-Joining networks were calculated with Network v.5.1.0.0 (Fluxus Technology Ltd., Suffolk, England) applying the default settings. In order to reduce unnecessary median vectors, the networks were post-processed with the maximum parsimony (MP) option. Networks were graphically prepared and supplemented by information on host species and geographic region in Network Publisher v.2.1.1.2 (Fluxus Technology Ltd.) and finalized in Adobe Illustrator CC v.19.0.0 (San Jose, California, USA). Co-infection was determined based on DNA sequencing and reflects the number of *Haemoproteus* lineages (1 = mono-infection, 2 = double-infection, and 3 = triple-infection).

### Statistical analysis

We explored whether individual body condition was associated with blood parasite parameters (i.e., infection intensity or co-infection level, models a and b) using linear models (LMs), and whether blood parasite parameters were associated with plumage phenotype using LM (model c) and a multinomial logistic regression (model d). Linear models were fit with the stats package (version 3.4.0) and the multinomial regression nnet package (7.3-12) (Ripley et al. 2016) in the R environment, version 3.4.4 (R Core Team 2018). All quantitative variables (body mass, tarsus length, and infection intensity) were scaled beforehand. Six individuals that showed no prevalence in the blood smear analysis (even if they were positives in the PCR screening) were excluded from all models featuring infection intensity. However, four of these were included in all models exploring the effects of co-infection on body condition and the effect of melanism expression on co-infection, because sequence data were available.

Previous research has shown that juvenile individuals are often suffering from higher infection intensities and are in lower body condition than adults (Sol et al. 2003). Therefore, before running the main analyses, we explored the influence of age-specific differences on individual body condition and infection intensity. Two linear models were fitted: one with body condition and a second with infection intensity (log-transformed to ensure normally distributed residuals) as response variables. Age was the key explanatory variable (immature or adult), and the length of the tarsus was added as a control variable in the model exploring body condition to control for individual variation in size. Due to a significant difference between immature and adult pigeons (see the “Results” section) and small sample size, immature individuals (*n* = 12) were excluded from the main models introduced below.

In the first set of the main analysis, we explored the effect of infection intensity (model a, *n* = 169) and co-infection (model b, *n* = 125) on individual condition and we fitted a linear model with body mass as the response variable and infection intensity (%) or co-infection of *Haemoproteus* spp. lineages (categorical variable, mono-, double-, or triple-infection) as explanatory variables. The length of the tarsus and the phenotype were also added as covariates to control for the individual size of the pigeon and to take the effect of different melanin expression levels into account. This approach was generally favored, rather than fitting residuals of a regression between body mass and tarsus as a body condition response in the model (García-Berthou 2001; Smith 1999).

In a second set of analysis, we explored the effect of plumage phenotype on blood parasite parameters, infection intensity (model c, *n* = 169), and co-infection (model d, *n* = 130). To do so, we fitted a linear model with log-transformed infection intensity as a response in order to fulfill the requirement of normally distributed residuals. In all linear models, residuals were visually inspected to ensure a normal distribution. Additionally, we explored whether pigeons of different phenotypes were infected by different numbers of blood parasites by fitting a multinomial logistic regression with the number of lineages as the response variable. Infection with only one parasite was the reference. *p* values were obtained by performing a Wald test (*z* test). In both models, the degree of melanism (phenotypes 1–4) was fitted as a continuous explanatory variable. As infection intensity might be influenced by multiple competing parasites (Clark et al. 2016), we fitted the co-infection variable as a fixed effect in explorative models a and c, and co-infection did not improve the model fit (by AIC) and was returned as not significant. It was thus removed from the final models.

## Results

### Haemosporidian parasite infection intensity and prevalence

Blood smears were prepared for 192 feral pigeons and scored for haemosporidian parasite infections. In the blood smear analysis, only six individuals, out of 192, were identified as uninfected (no parasites detected in 10,000 erythrocytes, 96.9% infection prevalence). However, four of these six birds did score positive in the PCR screening. Molecular screening could not be performed for one sample due to insufficient blood volume, and for a second one, the repeated PCR was negative. All positive samples were infected with *Haemoproteus* spp., whereas *Leucocytozoon* spp. and *Plasmodium* spp. were absent in our study population. The mean infection intensity of *Haemoproteus* spp. positive individuals was 1.3% (SD 2.9), ranging from 0.007 and 20.4% of erythrocytes being infected. The distribution of infection intensity was skewed, and the median was lower than the mean (median = 0.3%).

### Molecular analysis of haemosporidian parasites

**Fig. 1:**
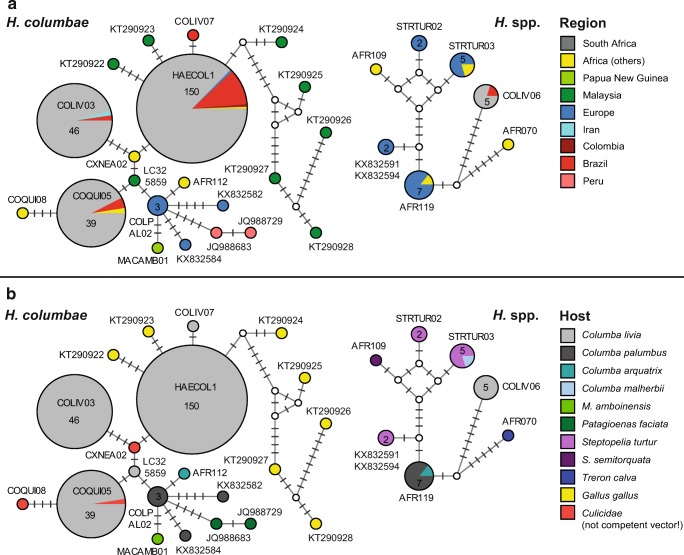
Median-joining haplotype networks of *cytb* sequences (476-bp) from the present study and GenBank. The size of the circles corresponds to the number of identical haplotypes. Bars on branches connecting the circles indicate the number of differences (substitutions) between haplotypes. Small white circles indicate median vectors. If available, the MalAvi lineage names are provided for each haplotype. Networks on the left side show haplotypes of *H. columbae* and related lineages, and networks on the right side show haplotypes of *Haemoproteus* spp. (COLIV06) and related lineages. **a** Information on host species. **b** Geographic distribution

Molecular screening for blood parasite lineages was performed on 144 DNA samples isolated from pigeon blood. PCRs for all but one sample were positive, and products were sequenced from 143 samples. Almost all samples contained lineages belonging to *H. columbae* (HAECOL1, COLIV03, COQUI05), which is a common parasite of pigeons, and only four samples contained another *Haemoproteus* lineage (COLIV06) not yet linked to a morphospecies. A high number of pigeons were infected with more than one parasite lineage. From the 143 PCR positive samples, 81 (56.6%) featured a single lineage, 51 (35.7%) two lineages, and 11 (15.7%) three lineages. The most common lineage HAECOL1 was found in 133 pigeons. We actually found two haplotypes matching 100% with HAECOL1, but differing in a single site at position 1003 of the *cytb*. The first variant (HAECOL1a) was found in 110 specimens and the second one (HAECOL1b) in 37 birds, and both haplotypes co-occurred in 16 individual pigeons. In the following, we refer to both of the latter haplotypes as lineage HAECOL1. The MalAvi lineages COLIV03 and COQUI05 were found in 44 and 36 samples, respectively. At the 478-bp DNA barcode section, HAECOL1 and COQUI05 differ from each other in three sites (0.6% uncorrected *p* distance), HAECOL1 and COLIV03 in four sites (0.8%), and COLIV03 and COQUI05 in five sites (1%). Only four pigeons were infected with the lineage COLIV06 belonging to an unidentified *Haemoproteus* species, differing from the *H. columbae* lineages by about 10%. The relationship of haplotypes found in the present study and already published data is displayed in Median-Joining networks (Fig. 1). All *cytb* sequences are available at NCBI GenBank under the accession numbers MN065190–MN065419.

### Relationship between parasite infection, plumage phenotype, age, and body condition

**Fig. 2:**
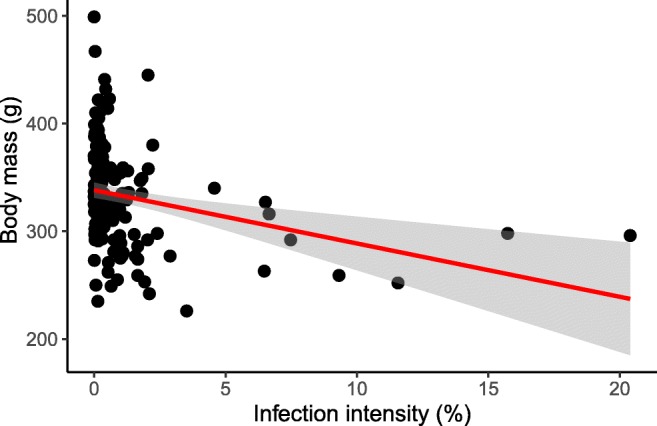
Relationship between individual body mass (g, raw values) and *Haemoproteus* spp. infection intensity (%). Each black dot represents one individual feral pigeon. Linear regression as a red line (adjusted *R*^2^ = 0.34). The standard error for the linear regression is shaded

Young pigeons (*n* = 12) had a significantly lower body condition (immature, 292.5 (SD 51.4); adult, 332.1 (SD 43.7); *χ*^2^ = 6.9, estimate = − 0.8 (SE 0.3), *n* = 174, *p* = 0.002) and higher infection intensity (immature, 5.7% (SD 5.7); adult, 1.0% (SD 2.4); *χ*^2^ = 53.2, estimate = 2.2 (SE 0.5), *n* = 174, *p* < 0.001). These young birds were excluded from the subsequent analysis. There was a significant (linear) negative relationship between infection intensity and body condition. Body mass decreased by 5.24 g with a 1% increase of infected red blood cell (Table 1, Fig. 2).

**Table 1:** Output of LMs showing effect sizes of blood parasite infection parameters on body condition (models a and b) and phenotype (i.e., degree of melanism) on infection intensity (model c)

Fixed effect	*df*	Estimate	SE	*χ*^2^	Pr(>*χ*^2^)
Model a: body mass ~ infection intensity + tarsus length + phenotype, *n* = 162					
Intercept	1	−0.039	0.170	0.031	0.821
Infection intensity	*1*	*−0.336*	*0.072*	*13.338*	*< 0.001*
Tarsus length	*1*	*0.523*	*0.066*	*38.972*	*< 0.001*
Phenotype	1	0.021	0.065	0.066	0.743
Model b: body mass ~ co-infection + tarsus length + phenotype, *n* = 125					
Intercept	1	0.269	0.687	0.070	0.697
Co-infection: 1 *Haemoproteus* spp. lineage	3	−0.150	0.684	0.228	0.918
Co-infection: 2 *Haemoproteus* spp. lineage	3	−0.090	0.682	0.228	0.918
Co-infection: 3 *Haemoproteus* spp. lineage	3	−0.244	0.720	0.228	0.918
Tarsus length	*1*	*0.448*	*0.067*	*20.481*	*< 0.001*
Phenotype	1	0.005	0.063	0.001	0.942
Model c: infection intensity ~ phenotype, *n* = 169					
Intercept	*1*	*−1.039*	*0.341*	*24.130*	*0.003*
Phenotype	1	−0.095	0.130	1.390	0.465

Sample size (*n*) for each model is given. Significant effects are indicated in italic

We found no relationship between the levels of co-infection of *Haemoproteus* spp. (mono-, double-, or triple-infection) and body condition (Table 1). We also found no relationship between plumage phenotype and infection intensity or co-infection levels (Table 1). Feral pigeons with a paler plumage had similar levels of infection intensity and co-infection as conspecifics with darker melanic plumage (Table 2).

**Table 2:** Output of a multinomial model showing effect sizes of phenotype (i.e., degree of melanism) on co-infection (model d)

Response	Reference category	*df*	Estimate	SE	*χ*^2^	Pr(>*χ*^2^)
Intercept: 2 *Haemoproteus* spp. lineages^1^	1	1	0.477	0.491	–	0.331
Intercept: 3 *Haemoproteus* spp. lineages^1^	*1*	*1*	*−2.970*	*1.200*	*–*	*0.013*
Co-infection: 2 *Haemoproteus* spp. lineages	1	3	−0.167	0.185	2.183	0.366
Co-Infection: 3 *Haemoproteus* spp. lineages	1	3	0.359	0.401	2.183	0.371

The sample size (*n*) available for this model is 130. Significant effects are indicated in italic

^1^ANOVA III was used to derive *χ*^2^ values, but it is not available for the intercept in multinomial logistic regression

## Discussion

Based on the blood smear analysis, the prevalence of *Haemoproteus* spp. was high in our study population with a 96.9% infection rate. Only six individuals did not show any infection with haemosporidian parasites based on the blood smear screening, but four of these tested positive through molecular screening. Screenings of blood smears often underestimate infection prevalence—especially when infection intensity is low—whereas PCR is more sensitive (Valkiūnas et al. 2008). Our results thus show that almost all feral pigeons sampled in suburban Cape Town were infected with *H. columbae*. This high rate is in line with results of a study conducted in Pretoria in the 1970s with 100% (Markus and Oosthuizen 1972), but much higher than previously reported in Cape Town in the 1990s with 72.7% infection rate (Earle and Little 1993). Both studies used only blood smear screenings and did not apply any PCR-based methods. Similarly high prevalence values were found in cities outside of South Africa, i.e., 97–100% in Madrid, Barcelona, and Granollers (Spain) (Sol et al. 2000; Vázquez et al. 2010); 100% in Sao Paulo (Brazil) (Chagas et al. 2016); or 100% in Hyattsville (Maryland, USA) (Knisley and Herman 1967). *Haemoproteus* spp. infection intensities of *C. livia* have been studied in other Sub-Saharan African countries with prevalence levels varying greatly both between and within countries. *Haemoproteus* spp. prevalence was high at 75–80% in Sebele (Botswana), Owerri (Nigeria), and Kampala (Uganda) (Dranzoa et al. 1999; Mushi et al. 2000; Opara et al. 2012); intermediate at 50% in Kano (Nigeria) (Karamba et al. 2012), and 37% in Morogoro (Tanzania) (Msoffe et al. 2010); and low at 15.2% in Zaria (Nigeria) (Owolabi et al. 2009). High prevalence levels and infection intensities of *Haemoproteus* spp. are not surprising, given that pigeons live and roost in flocks, making parasite transmission very easy (Johnston and Janiga 1995). Possibly, the high infection prevalence is depending on population density. Both *Haemoproteus* spp. prevalence and feral pigeon population density are generally high in suburban and urban studies (Chagas et al. 2016; Dranzoa et al. 1999; Karamba et al. 2012; Knisley and Herman 1967; Mushi et al. 2000; Opara et al. 2012; Sacchi et al. 2002; Sol et al. 2000; Vázquez et al. 2010). It can be assumed that these populations of feral pigeons are more likely to become infected by parasites and diseases because of their larger population densities. However, since natural populations of *C. livia* are poorly investigated, there is no data to compare with. *Haemoproteus columbae* does not infect non-columbid birds and thus is not a threat to other bird groups. However, breeding pigeons or native, naïve pigeon species are prone to become infected as the close proximity between wildlife, livestock, and pets could facilitate cross-species disease transmission in suburban and urban environments (Earle and Little 1993).

Although not much sequence data on avian haemosporidians of pigeons has been published so far, the four *cytb* lineages found in the present study seem to be common in feral pigeons. Exactly the same lineages were isolated from feral pigeons in the Sao Paolo Zoo (Chagas et al. 2016). The authors of the latter study confirmed that the lineages HAECOL1, COQUI05, and COLIV03 morphologically belong to *H. columbae*. The similarity in parasite lineage composition should be viewed in a historical context, since feral pigeons were introduced into both South Africa and South America by humans (Johnston and Janiga 1995), most likely together with their louse fly (Hippoboscidae) vectors. Apart from the four *H. columbae* lineages, only two other haplotypes are so far published for the feral pigeon: COLIV07 from Peru (Pacheco et al. 2017), differing in one site from HAECOL01, and an unnamed haplotype isolated from a pigeon in Japan (LC325859; published on GenBank only), differing in a single site from COQUI05. Related haplotypes have been isolated from common wood pigeon *Columba palumbus* in Europe (Dunn et al. 2017), African olive pigeon *Columba arquatrix* in Malawi (Lutz et al. 2015), Amboyna cuckoo-dove *Macropygia amboinensis* in Papua New Guinea (Beadell et al. 2004), band-tailed pigeon *Patagioenas fasciata* in Peru (JQ988683, JQ988729; published on GenBank only), and two mosquitoes (Culicidae, Diptera) in Cameroon (Njabo et al. 2011). Since the actual vector of *H. columbae* is the pigeon louse fly *P. canariensis* (Brooke 1981; Dean 2000; Sol et al. 2000), the parasites were probably in the blood meal of the mosquitoes. Interestingly, seven other related lineages (differing in 2 to 23 sites from HAECOL1) were found in both domestic and wild forms of the chicken *Gallus gallus* in Malaysia (Gimba et al., data published on GenBank only: KT290923–KT290928), which were not considered as hosts of *H. columbae* so far. Other than *H. columbae*, the second *Haemoproteus* species (lineage COLIV06) was found only in *Columbidae* birds. COLIV06 was elsewhere found in feral pigeons from Brazil, and several related lineages (differing in eight to 14 sites) were isolated in *C. palumbus* and European turtle-dove *Streptopelia turtur* in England (Dunn et al. 2017) and Spain (Drovetski et al. 2014); in red-eyed dove *Streptopelia semitorquata*, *C. arquatrix*, and African green pigeon *Treron calvus* in Malawi (Lutz et al. 2015); and in island bronze-naped pigeon *Columba malherbii* in Sao Tome and Principe (KT376899; published on GenBank only).

In our study, young pigeons had a significantly lower body condition and higher infection intensity than older individuals, a pattern often reported in different bird species (Sol et al. 2003). In adults, individual body condition was negatively correlated with infection intensity of *Haemoproteus*—implying that there may well be a physiological cost associated with being heavily infected. Parasites might drain energy from their host (Price et al. 1986), or infection could directly impair foraging activities (Marzal et al. 2005) and thus negatively affect body condition. To our knowledge, no correlation between *Haemoproteus* spp. and body condition in feral pigeons has been reported to date. The influence of different *Parahaemoproteus* parasites and infection intensity in non-columbids on body condition is frequently reported; however, these associations are not consistent. Negative effects of infection intensity on body condition have been found in American kestrel *Falco sparverius* (females only) (Dawson and Bortolotti 2000), but not in Eurasian kestrel *Falco tinnunculus* (Korpimäki et al. 1995) nor for European blackcap *Sylvia atricapilla* (during the first days after infection with *Haemoproteus belopolskyi*) (Valkiūnas et al. 2006). Furthermore, no significant negative correlations between infection intensity and body condition were documented in several other passerine species (Bennett et al. 1988; Granthon and Williams 2017), including during migration (correlation with body fat scores) (Ashford 1971). In the present study, co-infections with several *Haemoproteus* lineages did not affect the body condition more than infections with a single lineage only, which is in line with findings of Palinauskas et al. (2011) and Marzal et al. (2008). For future work, it would be interesting to investigate whether closely related lineages interact with each other. This interaction could be positive and favor co-infections, or it could be negative and closely related parasite lineages could compete within the host or within the vector. An example of a positive interaction was found in experimentally infected passerines. Two *Plasmodium* spp. acted synergetically during primary infections and were found to be highly virulent (Palinauskas et al. 2011). In a cross-genus study, microfilariae parasites were enhancing the co-infection of certain, but not all haemosporidians (Clark et al. 2016). In the present study, sequences were obtained by standard PCR and Sanger sequencing. Mixed infections were identified by the presence of wobble bases in the electropherograms, but we did not quantify the amount of each lineage in samples with mixed infections. It should be mentioned that with standard PCR and Sanger sequencing, parasites at much higher parasitemia (quantity) may be amplified preferentially, masking the fact that other parasites are present. In order to overcome this problem in future studies, deep amplicon sequencing would provide a possibility to accurately determine the percentage of two or more pathogen lineages in samples with mixed infections (Olmstead et al. 2019).

The feral pigeons caught for this study are part of a wild population in an area with large populations of native avian predator species (e.g. black sparrowhawk *Accipiter melanoleucus* (Martin et al. 2014; Rose et al. 2017); peregrine falcon *Falco peregrinus* (Jenkins 2000)), which may explain the absence of individuals with extremely high infection intensities of 50% or higher, since such birds might appear physically “sick” and might be rapidly removed from the system (Earle et al. 1993; Markus and Oosthuizen 1972). Most individuals sampled are likely exhibiting chronic infections rather than primary infections that are usually characterized by higher infection intensities (Asghar et al. 2012). Individuals during primary infections that show extreme infection intensities are more likely to die due to predation (Møller and Nielsen 2007; Temple 1987) or through the effects of the parasite themselves, like anemia (Earle et al. 1993). Our results show that *H. columbae* infection intensity has negative effects on a body condition which could lower individual fitness (Chastel et al. 1995; Cichoiń et al. 1998). However, our study does not include any long-term effects on individual survival, mate choice, or reproductive success and no such studies have been conducted to date on the *H. columbae*-*C. livia* parasite-host system. However, long-term fitness correlations have been studied in other species, for example female Eurasian kestrels mating with males infected with *Haemoproteus* (*Parahaemoproteus*) *tinnunculi* laid later and smaller clutches than females mating with uninfected males (Korpimäki et al. 1995), thus suggesting that *Haemoproteus* spp. infections play a role in mate choice. Prevalence of *Haemoproteus* (*Parahaemoproteus*) *payevskyi* in female great reed warblers *Acrocephalus arundinaceus* led to a smaller number of fledged offspring (Asghar et al. 2011), and high infection intensity of *Haemoproteus* spp. negatively influenced the return rates of female American kestrels, implying that infection reduced survival rates (Dawson and Bortolotti 2000). On the contrary, white-crowned sparrow *Zonotrichia leucophrys oriantha* females infected with *Haemoproteus* (*Parahaemoproteus*) *beckeri* had higher survival rates and about twice as high lifetime reproductive success than uninfected females (Zylberberg et al. 2015).

Interestingly, other avian malaria parasites like *Leucocytozoon* spp. and *Plasmodium* spp. were absent in our study population. *Leucocytozoon* spp. have been reported in two male *C. livia* in South Africa in the 1990s, one from the southwestern Cape and one from Stromberg, Eastern Cape (Earle and Little 1993). The absence of *Leucocytozoon* spp. and *Plasmodium* spp. in feral pigeons has also been reported from Sao Paulo (Brazil) (Chagas et al. 2016) and Santiago (Chile) (Toro et al. 2010); however, in the latter study, no *Haemoproteus* spp. were found in the feral pigeon population either. *Leucocytozoon* spp. and *Plasmodium* spp. use different dipteran vectors than *Haemoproteus* spp. with *Leucocytozoon* spp. being mainly transmitted by blackflies (*Simulium* spp., Simuliidae, Diptera) and *Plasmodium* spp. by mosquitoes (Culicidae, Diptera) (Njabo et al. 2009; Russell and Mohan 1942; Ventim et al. 2012). Blackflies need running, fast-flowing streams (Rivers-Moore et al. 2007), which might be absent in highly urbanized areas in Cape Town, as suggested in a haemosporidian parasite study in black sparrowhawks (Suri et al. 2017). In that study, prevalence of *Leucocytozoon* spp. significantly decreased with a higher degree of urbanization; however, the prevalence for *Haemoproteus* spp. was similar along the urban gradient (Suri et al. 2017). The absence of vectors seems unlikely to cause the absence of *Plasmodium* in this study, and feral pigeons might not be native hosts for *Plasmodium* parasites (but see the experimentally infected *C. livia* with *Plasmodium* (*Giovannolaia*) *gabaldoni* and *Plasmodium* (*Novyella*) *columbae*) (Valkiūnas 2004). Generally, *Plasmodium* species were reported only rarely in wild feral pigeons (see exceptions, i.e., Gupta et al. 2011; Natala et al. 2009; Opara et al. 2012), but none of these records was confirmed by molecular screening methods and *Haemoproteus* and *Plasmodium* can easily be confused depending on the development stage of the parasite and the quality of the blood slide (Valkiūnas 2004).

Lastly, no effect of eumelanism on the infection intensity was found. Higher levels of eumelanism are hypothesized to have beneficial effects on the immune system (Ducrest et al. 2008) and have been shown to be correlated with improved immunocompetence in other species (Baeckens and Van Damme 2018; Figuerola et al. 1999; Galeotti and Sacchi 2003; Galván et al. 2010; Roulin et al. 2001). Lei et al. (2013) found for the color-polymorphic black sparrowhawk that darker individuals showed lower infection intensity. In the barn owl *Tyto alba*, individuals with more melanistic spots in their plumage had a smaller bursa of Fabricius, suggesting that they were less parasitized (Roulin et al. 2001). In feral pigeons, darker individuals are assumed to show stronger immune response, but blood parasite intensity was independent of melanism in a study by Jacquin et al. (2011), a result that is in line with our finding. Differences of blood parasite intensity and melanism were found only along an urban gradient, and only when completely white individuals were considered (Jacquin et al. 2013), which were largely absent in our study system.

To conclude, our study represents the largest dataset to date that combines sequences of mitochondrial *cytochrome b* (*cytb*) of haemosporidian parasites in feral pigeons, and provides evidence for a negative association between host body condition and parasite infection with *Haemoproteus* spp. However, it would be interesting to understand the long-term fitness consequences of infection by *Haemoproteus* spp. on feral pigeons, a species that is closely associated with humans, and has been introduced to several cities across the globe. This is especially the case, given that there is the potential for it to introduce its parasites to naïve native avifauna.

## Data Availability

The dataset is available as electronic supplement (Appendix Table S1), and *cytochrome b* sequences are available on NCBI GenBank under the accession numbers MN065190–MN065419.

## Appendix

**Table S1:** Individual sample list of *C. livia* that were collected in Cape Town as part of this study

Pigeon ID	Date	Age	Tarsus (mm)	Body mass (g)	Plumage	Infection intensity (%)	Prevalence	HAECOL1	COLIV03	COQUI05	COQUI06	Co-infection
Cliv01	07/05/2018	Adult	37.00	356	2	1.28	1					
Cliv02	07/05/2018	Adult	40.50	330	3	1.19	1					
Cliv03	07/05/2018	Adult	35.10	297	2	1.52	1	1	1	0	0	1
Cliv04	07/05/2018	Adult	36.80	399	1	0.01	1	1	1	0	0	1
Cliv05	07/05/2018	Adult	40.60	414	2	0.52	1					
Cliv06	08/05/2018	Adult	40.00	422	1	0.16	1	1	0	0	0	0
Cliv07	08/05/2018	Adult	38.30	332	2	0.01	1	1	1	0	0	0
Cliv08	08/05/2018	Adult	40.70	467	2	0.04	1					
Cliv09	08/05/2018	Adult	37.20	385	2	0.14	1					
Cliv10	09/05/2018	Adult	39.00	410	2	0.06	1	1	0	0	0	0
Cliv11	09/05/2018	Adult	36.70	320	3	0.97	1					
Cliv12	09/05/2018	Adult	35.60	259	1	1.66	1					
Cliv13	09/05/2018	Adult	40.50	423	3	0.58	1					
Cliv14	09/05/2018	Adult	40.30	445	1	2.05	1	1	0	0	0	0
Cliv15	10/05/2018	Adult	38.30	332	3	0.00	0	1	0	0	0	0
Cliv16	10/05/2018	Adult	38.50	235	3	0.14	1					
Cliv17	10/05/2018	Immature	37.20	199	1	3.50	1					
Cliv18	10/05/2018	Adult	36.00	250	2	0.07	1	1	0	0	0	0
Cliv19	10/05/2018	Adult	37.10	286	0	0.79	1					
Cliv20	11/05/2018	Adult	33.20	298	3	2.40	1	1	0	0	0	0
Cliv21	11/05/2018	Adult	34.00	242	2	2.10	1					
Cliv22	12/05/2018	Adult	36.60	292	2	0.04	1					
Cliv23	12/05/2018	Adult	32.50	337	4	0.00	0	1	1	0	0	1
Cliv24	12/05/2018	Adult	39.00	316	3	6.66	1	1	0	0	0	1
Cliv25	14/05/2018	Adult	35.70	293	4	0.31	1					
Cliv26	14/05/2018	Adult	34.70	253	1	1.92	1					
Cliv27	14/05/2018	Immature	29.50	238	1	0.75	1	1	1	1	0	1
Cliv28	14/05/2018	Adult	33.30	319	3	0.03	1	1	0	0	0	0
Cliv29	14/05/2018	Adult	31.80	262	2	0.53	1	1	1	0	0	1
Cliv30	14/05/2018	Adult	37.10	327	3	0.20	1	0	1	1	0	1
Cliv31	14/05/2018	Adult	35.50	298	4	15.74	1	1	1	0	0	1
Cliv32	15/05/2018	Immature	38.00	312	2	8.49	1	0	1	0	0	0
Cliv33	14/05/2018	Adult	39.50	355	3	0.35	1	1	1	0	0	1
Cliv34	14/05/2018	Adult	38.10	394	3	0.16	1	1	0	0	0	0
Cliv35	14/05/2018	Adult	34.60	327	3	6.52	1					
Cliv36	17/05/2018	Adult	36.50	328	3	0.03	1					
Cliv37	17/05/2018	Adult	34.90	277	2	1.56	1	1	1	1	0	1
Cliv38	17/05/2018	Adult	36.60	441	2	0.40	1					
Cliv39	17/05/2018	Adult	39.00	388	2	0.01	1	1	0	1	0	1
Cliv40	17/05/2018	Adult	40.50	371	2	0.16	1	1	0	0	0	0
Cliv41	17/05/2018	Adult	37.10	322	2	0.82	1	1	0	0	0	0
Cliv42	16/06/2018	Adult	34.70		3	0.01	1	1	0	0	0	0
Cliv43	16/06/2018	Adult	38.70	320	2	0.47	1	0	1	0	0	0
Cliv44	16/06/2018	Adult	36.00		2	0.31	1	1	0	0	0	0
Cliv45	16/06/2018	Adult	39.17	368	2	0.07	1	1	0	0	0	0
Cliv46	16/06/2018	Adult	34.48		1	0.76	1	1	0	0	0	0
Cliv47	16/06/2018	Adult	38.66	362	2	0.15	1	1	0	1	0	1
Cliv48	16/06/2018	Adult	30.99		4	0.00	0	1	1	0	0	1
Cliv49	16/06/2018	Adult	37.28	311	4			1	1	1	0	1
Cliv50	16/06/2018	Adult			4	0.07	1					
Cliv51	16/06/2018	Adult	38.16	334	3	0.40	1	1	0	0	0	0
Cliv52	16/06/2018	Adult	38.75		2	0.08	1	1	1	0	0	1
Cliv53	16/06/2018	Adult	31.58	306	3	0.08	1	1	0	0	0	0
Cliv54	17/05/2018	Adult	38.10	346		0.02	1	0	0	1	0	0
Cliv55	17/05/2018	Adult	35.50	324	3	0.40	1	1	1	0	0	1
Cliv56	17/05/2018	Adult	38.50	329	3	1.23	1	1	1	0	0	1
Cliv57	17/05/2018	Adult	39.60	335	4	1.12	1					
Cliv58	17/05/2018	Adult	34.20	334	3	0.05	1	1	0	0	0	0
Cliv59	17/05/2018	Adult	35.00	318	4	0.24	1	1	0	0	0	0
Cliv60	17/05/2018	Adult	34.60	275	3	1.00	1	1	0	0	0	1
Cliv61	17/05/2018	Adult	38.30	340	1	0.18	1	1	0	1	0	1
Cliv62	17/05/2018	Adult	36.40	290		1.35	1	1	0	0	0	0
Cliv63	17/05/2018	Adult	37.70	335	2	1.82	1	1	1	1	0	1
Cliv64	17/05/2018	Adult	39.20	354	1	0.99	1	1	1	0	0	1
Cliv65	17/05/2018	Adult	39.20	385	3	0.11	1	1	1	1	0	1
Cliv66	16/06/2018	Adult	37.63	367	3	0.05	1	1	0	0	0	0
Cliv67	16/06/2018	Adult	35.04	371	1	0.13	1	1	0	0	0	1
Cliv68	16/06/2018	Adult	39.29	391	1	0.03	1	1	1	0	0	1
Cliv69	16/06/2018	Adult	35.42	357	2	0.53	1	1	0	1	0	1
Cliv70	16/06/2018	Adult	38.66	411	4	0.23	1	1	0	1	0	1
Cliv71	16/06/2018	Adult	35.24	328		0.21	1	1	0	0	0	0
Cliv72	16/06/2018	Adult	35.87	321	1	0.16	1	1	0	0	0	0
Cliv73	16/06/2018	Adult	34.20	313	4	1.19	1	1	1	1	0	1
Cliv74	16/06/2018	Adult	35.60	281	3	0.75	1	1	0	1	0	1
Cliv75	16/06/2018	Adult	36.17	302	4	0.03	1	1	1	0	0	1
Cliv76	16/06/2018	Adult	40.80	358	2	0.13	1	1	0	0	0	0
Cliv77	16/06/2018	Adult	37.94	381	4	0.30	1	1	1	0	0	1
Cliv78	19/05/2018	Adult	38.80	303	3	0.24	1	1	1	0	0	1
Cliv79	19/05/2018	Adult	35.00	289	3	1.02	1	1	0	0	0	0
Cliv80	19/05/2018	Adult	39.00	358	3	2.06	1	1	0	1	0	1
Cliv81	19/05/2018	Adult	36.50	315	2	0.86	1	1	1	0	0	1
Cliv82	19/05/2018	Adult	35.10	274	3	1.66	1	1	0	1	0	1
Cliv83	19/05/2018	Adult	37.20	340	2	4.57	1					
Cliv84	19/05/2018	Adult	33.10	286	3	1.66	1	1	0	0	0	0
Cliv85	19/05/2018	Adult	39.20	370	1	0.00	0	1	1	0	0	1
Cliv86	19/05/2018	Adult	34.90	302	3	0.29	1					
Cliv87	19/05/2018	Adult	38.00	306		1.89	1	1	0	0	0	0
Cliv88	19/05/2018	Adult	38.60	343	3	0.01	1					
Cliv89	19/05/2018	Adult	36.10	315	3	0.19	1	1	1	0	0	1
Cliv90	16/06/2018	Adult	36.71	356	1	0.35	1	1	1	0	0	1
Cliv91	16/06/2018	Adult	36.59	344	3	0.07	1	1	1	0	0	1
Cliv92	16/06/2018	Adult	39.54	341	4	0.11	1					
Cliv93	16/06/2018	Adult	39.81	396	2	0.12	1	1	0	0	0	0
Cliv94	19/05/2018	Adult	39.80	361	1	0.25	1	1	0	0	0	0
Cliv95	19/05/2018	Adult	35.40	387	4	0.20	1	1	0	1	0	1
Cliv96	19/05/2018	Adult	33.20	297	1	0.24	1	1	1	0	0	1
Cliv97	19/05/2018	Adult	40.00	380	3	2.23	1	1	0	0	0	0
Cliv98	19/05/2018	Adult	37.20	302	2	0.09	1	1	0	0	0	0
Cliv99	19/05/2018	Adult	36.00	336	2	0.18	1	1	0	0	0	1
Cliv100	19/05/2018	Adult	36.20	348	2	0.12	1					
Cliv101	19/05/2018	Adult	33.60	322	3	0.06	1	1	1	0	0	1
Cliv102	19/05/2018	Adult	39.40	336	3	0.03	1	1	0	0	0	0
Cliv103	19/05/2018	Adult	39.00	359	3	0.62	1	1	0	0	0	0
Cliv104	19/05/2018	Adult	38.50	317	2	0.43	1	1	0	0	0	0
Cliv105	19/05/2018	Adult	34.70	378	3	0.40	1	1	0	0	0	0
Cliv106	16/06/2018	Adult	38.43	379	3	0.08	1	1	0	0	0	0
Cliv107	21/06/2018	Immature	38.67	327	2	0.41	1	1	0	0	0	0
Cliv108	16/06/2018	Adult	40.25	326	2	0.07	1	0	1	1	0	1
Cliv109	16/06/2018	Adult	34.70	364	3	0.33	1	1	0	1	0	1
Cliv110	21/06/2018	Adult	39.95	327	4	0.36	1	1	0	0	0	0
Cliv111	16/06/2018	Adult	39.86	339	3	0.07	1	1	0	0	0	0
Cliv112	21/06/2018	Adult	37.77	292	1	7.48	1	1	0	0	0	1
Cliv113	21/06/2018	Immature	37.62	252	3	17.75	1	1	1	1	0	1
Cliv114	21/06/2018	Adult	37.30	339	3	0.09	1	1	0	0	0	1
Cliv115	16/06/2018	Adult	35.99	340	3	0.16	1	1	0	1	0	1
Cliv116	21/06/2018	Adult	32.41	292	3	2.03	1					
Cliv117	21/06/2018	Adult	33.89	292	2	0.75	1	1	0	0	0	0
Cliv118	16/06/2018	Adult	38.14	328	4	0.55	1	1	0	0	0	0
Cliv119	16/06/2018	Adult	34.46	292	1	0.06	1					
Cliv120	21/06/2018	Adult	32.57	249	1	0.65	1					
Cliv121	16/06/2018	Adult	38.18	296	2	0.97	1	1	0	0	0	0
Cliv122	19/05/2018	Adult	35.50	277	2	2.89	1					
Cliv123	19/05/2018	Adult	35.50	307	2	0.05	1	1	0	0	0	1
Cliv124	19/05/2018	Adult	36.40	339	3	0.21	1	1	1	0	0	1
Cliv125	19/05/2018	Adult	38.10	331	3	0.30	1	1	0	0	0	1
Cliv126	19/05/2018	Adult	34.30	277	2	1.06	1	1	0	1	0	1
Cliv127	19/05/2018	Immature	36.30	296	1	12.23	1	0	0	1	0	0
Cliv128	19/05/2018	Adult	35.00	367	4	0.17	1	1	0	1	0	1
Cliv129	19/05/2018	Adult	39.30	345	2	0.29	1					
Cliv130	19/05/2018	Adult	40.50	318	3	0.01	1					
Cliv131	19/05/2018	Adult	36.00	349	1	1.82	1	1	1	0	0	1
Cliv132	19/05/2018	Adult	36.70	346	3	0.12	1					
Cliv133	19/05/2018	Immature	35.60	284	3	8.10	1	1	0	1	0	1
Cliv134	21/06/2018	Adult	36.87	333	1	0.27	1	1	0	0	0	1
Cliv135	21/06/2018	Adult	35.57	273	2	0.01	1	0	1	0	0	0
Cliv136	21/06/2018	Adult	38.49	366	3			1	0	0	0	0
Cliv137	21/06/2018	Adult	37.55	336	1	1.31	1	1	0	1	0	1
Cliv138	21/06/2018	Adult	36.83	388	1	0.08	1	1	0	0	0	1
Cliv139	21/06/2018	Adult	37.64	352	2	0.14	1	1	0	0	0	0
Cliv140	21/06/2018	Adult	37.29	340	2	0.03	1	1	0	0	0	1
Cliv141	21/06/2018	Adult	40.55	390	3	0.12	1	1	0	0	0	1
Cliv142	21/06/2018	Adult	38.08	352	3	0.48	1	1	0	0	0	1
Cliv143	21/06/2018	Immature	37.22	383	1	0.21	1	1	0	1	0	1
Cliv144	21/06/2018	Adult	38.87	343	1	0.02	1					
Cliv145	21/06/2018	Adult	38.66	348	4	0.78	1					
Cliv146	21/06/2018	Adult	36.11	348	2	0.24	1	1	0	1	1	1
Cliv147	21/06/2018	Adult	37.94		1	0.15	1	1	0	0	1	1
Cliv148	21/06/2018	Adult	38.67	335	2	1.06	1	1	1	1	0	1
Cliv149	21/06/2018	Adult	37.75	338	4			1	0	0	0	0
Cliv150	23/04/2018	Adult	41.00	296	3	20.40	1					
Cliv151	23/04/2018	Adult	35.00	226	2	3.52	1					
Cliv152	23/04/2018	Adult	39.00	259	2	9.32	1					
Cliv153	19/05/2018	Adult	38.20	310	1	0.24	1	1	0	0	0	1
Cliv154	19/05/2018	Adult	33.20	297	4	0.15	1	1	0	0	0	0
Cliv155	19/05/2018	Immature	38.20	271	3	8.78	1	1	1	0	0	1
Cliv156	19/05/2018	Adult	36.40	291	1	0.15	1	0	0	0	1	0
Cliv157	19/05/2018	Adult	34.80	299	2	0.07	1	1	0	0	0	0
Cliv158	19/05/2018	Adult	41.20	348	2	0.11	1	1	0	0	0	0
Cliv159	19/05/2018	Adult	38.30	335	4	0.36	1					
Cliv160	19/05/2018	Adult	37.10	328	3	0.88	1	1	0	1	0	1
Cliv161	19/05/2018	Adult	36.40	280	2	1.11	1	0	1	1	0	1
Cliv162	19/05/2018	Adult	36.40	297	3	0.02	1					
Cliv163	19/05/2018	Adult	39.20	351	3	0.14	1					
Cliv164	19/05/2018	Immature	36.20	286	1	0.55	1					
Cliv165	19/05/2018	Adult	33.70	310	1	0.71	1	1	0	0	0	0
Cliv166	19/05/2018	Adult	38.70		3	0.21	1					
Cliv167	19/05/2018	Adult	36.80	314	2	0.13	1	1	0	0	0	0
Cliv168	21/06/2018	Adult	35.75	348	1	0.14	1	1	0	1	0	1
Cliv169	21/06/2018	Adult	35.72	317	4	0.98	1	1	0	0	0	0
Cliv170	21/06/2018	Adult	36.64	347	3	1.75	1	1	0	0	0	0
Cliv171	21/06/2018	Adult	34.49	317	3	0.13	1	1	0	1	0	1
Cliv172	21/06/2018	Adult	37.98	372	4	0.23	1	1	0	0	0	0
Cliv173	21/06/2018	Adult	34.19	328	1	0.03	1	1	1	0	0	1
Cliv174	21/06/2018	Adult	40.61	333	4	0.01	1	1	1	0	0	1
Cliv175	21/06/2018	Adult	35.08	325	2	0.00	0					0
Cliv176	21/06/2018	Adult	38.45	342	2	0.19	1	1	0	0	0	0
Cliv177	21/06/2018	Adult	36.64	369	4	0.32	1	1	0	0	0	0
Cliv178	21/06/2018	Adult	36.13	367	3	0.01	1	0	0	1	0	0
Cliv179	21/06/2018	Adult	36.20	340	3	0.14	1	1	1	0	0	1
Cliv180	21/06/2018	Adult	35.58	353	2	0.46	1	1	0	0	0	0
Cliv181	21/06/2018	Adult	38.87	354	4	0.05	1	1	0	1	1	1
Cliv182	21/06/2018	Adult	37.46	359	2	1.10	1	1	0	0	0	1
Cliv183	21/06/2018	Adult	36.30	304	1	0.38	1	1	0	0	0	0
Cliv184	21/06/2018	Adult	36.79	335	4	0.31	1	1	0	0	0	0
Cliv185	21/06/2018	Adult	38.23	313	4	0.13	1	1	0	0	0	0
Cliv186	21/06/2018	Immature	38.13	296	1	7.32	1	1	1	1	0	1
Cliv187	24/04/2018	Adult	36.50	271	3	0.54	1					
Cliv188	24/04/2018	Adult	39.00	252	1	11.56	1					
Cliv189	24/04/2018	Adult	36.00	255	3	0.89	1					
Cliv190	24/04/2018	Adult	36.00	263	1	6.47	1					
Cliv191	25/04/2018	Adult	40.00	327	1	0.71	1					
Cliv192	25/04/2018	Adult	47.00	499	1	0.00	0					
Cliv193	25/04/2018	Adult	41.00	432	2	0.44	1					
Cliv194	25/04/2018	Immature	38.00	366	3	0.73	1					
Cliv195	25/04/2018	Adult	38.00	405	2	0.18	1					

The table lists individual details (age, body morphometrics) and its parasite inflection parameters. Pigeon ID is the individual identifier given each individual. Date refers to the date of collection and age to the age class (adult > 6 months, immature < 6 months). Body morphometrics are tarsus length (“tarsus”) and weight (“body mass”). The phenotype score (0–4) can be found in the “plumage” column and was obtained following the protocol established by Johnston and Janiga (1995) and Jacquin et al. (2011). Infection intensity is given in percentages. The prevalence score is binary (either 0 for uninfected or 1 for infected). Both infection intensity and prevalence are based on microscopy analysis. Additional binary codes (0 for not present, 1 for present) are given for the *Haemoproteus* spp. lineages detected in this study. The “co-infection” column refers whether multiple lineages are present or not
